# Using individual stated-preferences to optimize HIV self-testing service delivery among men who have sex with men (MSM) in Malaysia: results from a conjoint-based analysis

**DOI:** 10.1186/s12889-020-09832-w

**Published:** 2020-11-25

**Authors:** Roman Shrestha, Haridah Alias, Li P. Wong, Frederick L. Altice, Sin H. Lim

**Affiliations:** 1 Section of Infectious Disease, Department of Internal Medicine, Yale School of Medicine, 135 College Street, Suite 323, New Haven, CT 06510 USA; 2 Centre of Excellence for Research in AIDS (CERiA), Faculty of Medicine, University of Malaya, Kuala Lumpur, Malaysia; 3 Department of Social & Preventive Medicine, Faculty of Medicine, University of Malaya, Kuala Lumpur, Malaysia

**Keywords:** HIV, HIV self-testing, Men who have sex with men, Implementation science, Conjoint analysis, Malaysia

## Abstract

**Background:**

HIV self-testing (HIVST) has the potential to improve HIV testing uptake and frequency for key populations like MSM who experience multiple barriers accessing clinic-based HIV testing. In the absence of HIVST in Malaysia, there is no guidance to inform HIVST delivery. This study investigated the acceptability of HIVST and preferences about the HIVST service delivery approaches using a standardized stated preference method.

**Methods:**

A cross-sectional online survey conducted between January and April 2019 assessed the interest in HIVST in 544 MSM in Malaysia. Participants ranked eight hypothetical HIVST service delivery program elements with varied combinations of six, two-level HIVST service delivery program attributes (cost, privacy, accuracy, kit collection site, kit type, and testing support). SPSS conjoint procedure was used to estimate the relative importance of each attribute and preference across eight possible HIVST service delivery programs.

**Results:**

Overall, 70.4% had previously tested for HIV, and of those, 64.0% had done so in the past 6 months (45.0% of all participants). Of all the participants, 25.2% reported having used HIVST previously. The acceptability for HIVST service delivery models ranged from 44.9 to 77.1%, with mean acceptability of 56.2% across the eight hypothetical HIVST distribution scenarios. The HIVST service delivery scenario with the highest acceptability had the following attributes: no cost (free), anonymity (name not required), 99–100% accuracy, home-delivered, fingerstick, and testing support using telephone hotline or texting. HIVST cost was the most important attribute (relative importance score: RIS = 19.30) associated with acceptability, followed by anonymity (RIS = 18.41), accuracy (RIS = 17.33), kit delivery (RIS = 16.99), fingerstick kit (RIS = 15.86), and support (RIS = 12.08).

**Conclusions:**

Acceptability for HIVST in Malaysian MSM was high but differed markedly by a number of HIVST delivery scenarios and attributes. These findings could be relevant as the Malaysian Ministry of Health is in the process of developing a regulatory framework for ensuring the quality of kits, as well as policies supporting safe use while broader implementation under national AIDS programs.

## Background

HIV testing is the first step towards achieving UNAID’s 95–95-95 targets [1] as well as for bridging the HIV prevention gap to scale-up pre-exposure prophylaxis (PrEP) in key populations [2]. For key population groups like men who have sex with men (MSM), however, HIV testing uptake is often low due to multilevel factors like fear, stigma and discrimination, criminalization, and disclosure of sexual/gender identity, lack of anonymity, and concerns about confidentiality [3–8]. In Malaysia, a middle-income country with a Muslim majority, same-sex behaviors are criminalized in both secular and Sharia laws, resulting in high levels of stigma and de facto discrimination. Consequently, MSM in Malaysia inadequately test, which has thwarted scale-up of effective HIV testing, prevention, and treatment programs for the lesbian, gay, bisexual, and transgender (LGBT) community.

Sexual health programs have mainly focused on mandatory HIV testing among Muslim couples planning to marry. Consequently, HIV prevention and treatment programs are inadequately designed for MSM, who now account for most new HIV infections in Malaysia [9]. With only 61.6% of Malaysian MSM having ever tested for HIV and less than half (44.5%) tested in the past 12 months [10], innovations in HIV testing are needed. In an effort to address gaps in delivering HIV testing services, to achieve national and global testing targets, the World Health Organization (WHO) recommends and guides HIV self-testing (HIVST) to supplement traditional facility and community outreach testing [11]. HIVST offers an alternative to other testing strategies, including reaching new MSM and increase testing frequency to facilitate early HIV detection and treatment and prevent new infections [12, 13]. HIVST increases convenience, privacy, and confidentiality, but its uptake may be limited by cost for some MSM, particularly in Malaysia, concerned with legal constraints and face high rates of stigma and discrimination.

While HIVST research has increased globally, no empirical studies have been conducted in Malaysia. HIVST is currently not licensed in Malaysia, and the country does not have any formal regulation of HIVST kits yet. However, unregulated HIVST kits are available for purchase at several pharmacies nationwide and are also widely available through the internet (RM20 = ~ USD5). As the Malaysian Ministry of Health develops its national HIVST policy to expand HIV testing in key populations, empirical evidence to guide nation-wide HIVST strategy is critical. Specifically, MSM are heterogeneous in terms of their preferences about HIV testing, and as HIVST becomes an option for them, it is crucial to understand elements that are important for HIVST programs that have the potential for jumpstarting the HIV treatment and prevention cascades [14]. To better understand preferences about HIVST in MSM, we examined the acceptability of HIVST and relative preferences about the elements of HIVST delivery, using a standardized stated preference method (conjoint-based analysis; CBA) to assess how Malaysian MSM value various attributes of hypothetical HIVST service delivery models.

## Methods

### Study design and participants

In preparation for introducing HIVST, a cross-sectional online survey to assess the willingness to adopt HIVST in Malaysia was conducted between January and April 2019. Individuals are eligible if they: i) are aged 18 years or older; ii) are Malaysian citizens; iii) are biologically male who reported having sex with other men in the past 12 months or being a transgender woman (male sex assigned at birth and reporting gender identity as female); and iv) self-reported HIV-negative or HIV status unknown. The study was initially planned to include both MSM and TGW in Malaysia. However, we were unable to recruit enough TGW to include them in our analysis (*n* = 6). We therefore excluded 6 TGW participants from the analysis to ensure the generalizability of the findings to the Malaysian MSM community.

### Study procedures

Participants were recruited using a combination of both online and offline recruitment strategies. Specifically, participants were recruited through social media popular among MSM in Malaysia (e.g., Grindr, Hornet), AIDS and LGBT-friendly NGO social media profiles, and peer referrals. A standardized script was used to message prospective participants. Those interested in participating received a web link directing them to the online survey hosted by Qualtrics, where eligibility criteria were specified. Each eligible participant voluntarily completed the IRB-approved online consent form before initiating the survey. On average, it took 12 min for the participants to complete the anonymous online survey. Participation in the survey was voluntary, and participants were not paid for completing the survey. The study protocol and the consent form were approved by the University of Malaya Research Ethics Committee (UMREC).

### Study measures

#### Participant characteristics

included age, gender identity, ethnicity, relationship status, educational attainment, employment, and income. Participants self-reported engagement in HIV risk behaviors (i.e., sexual- and drug-related factors), history of HIV testing practices, and stigma and discrimination related to sexual orientation. Participants were also asked about their awareness of HIVST and previous use of HIVST.

#### Conjoint-based analysis (CBA)

Stated preference methods are increasingly being used to enable policymakers to quantify the values that key populations attach to each attribute of service delivery and to determine which combination of features is likely to result in optimal health outcomes in this stigmatized and hard-to-reach group [15].

We used a full-profile CBA design to quantify the importance of various attributes of the HIVST program. CBA is an established statistical technique of assessing consumer preferences in market research, and it is increasingly being applied to evaluate health-care intervention [16–18].

We identified six dichotomous attributes relevant to HIVST program, that included: cost (*free* vs. *pay some money*); privacy (*name not required* vs. *name required*); accuracy (*99–100%* vs. *95%*); kit collection site (*home-delivered* vs. *at a pharmacy*); kit type (*fingerstick* vs. *oral swab*); and testing support (*telephone hotline/texting* vs. *online videos*). The array of attributes and their dichotomous values were developed based on inputs from experts working with the target populations and published research on HIVST acceptability [19–24]. The six dichotomous HIVST attributes yielded 64 (2^6^ = 64) different HIVST distribution conjoints (scenarios). We employed a fractional factorial orthogonal design [25] to generate a subset of representative scenarios (each attribute/level combination appears the same number of times) given the high burden on participants to rate every scenario. This method enabled us to reduce the number of HIVST service delivery scenarios from 64 to 8.

**Fig. 1:**
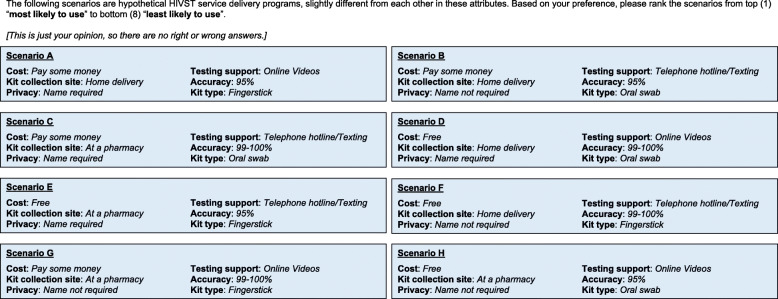
Example of full-profile conjoint task (hypothetical HIV self-testing program scenarios)

The hypothetical HIVST distribution scenarios were presented simultaneously after the participants were provided with a brief description of the HIVST attributes and their values. Participants were then asked to rank the eight scenarios in terms of acceptability from 1 (“*most likely to use*”) to 8 (“*least likely to use*”). None of the scenarios could share the same value (Fig. 1). The scenarios were presented randomly using a randomization feature of Qualtrics to prevent order effect bias.

### Statistical analysis

Among the 550 participants recruited, data for 544 were analyzed (6 participants were TGW and excluded from the analysis). We computed descriptive statistics, including frequencies and percentages for categorical variables and means and standard deviations for continuous variables. We used the chi-square test and t-test to compare the frequencies (or means) for those willing and unwilling to use HIVST. We then used CBA to explore the acceptability of HIVST delivery scenarios. The acceptability of each HIVST distribution scenario was derived by averaging individual HIVST distribution model acceptability ratings across respondents. Ratings from each HIVST distribution model was transformed into a 0–100 scale, whereby “*most likely to use*” =100 and “least likely to use” =0. We employed the SPSS *Conjoint* procedure to generate marginal utility scores (MUS; a measure of preference of each factor level) for each attribute level [26] and the relative importance score (RIS; a measure of the importance of the attribute) [27, 28].

## Results

### Participant characteristics

Table 1 provides a summary of the characteristics of 544 participants. Most participants were single (75.2%), university graduates (87.1%), and currently employed (71.5%). In terms of sexual behaviors, 84.0% of participants reported having anal sex with another man in the past six months, with 79.6% involved in condomless sex. Overall, 70.4% had previously tested for HIV, and of those, 64.0% had done so in the past 6 months (45.0% of all participants). Of all the participants, 25.2% reported having used HIVST previously. Most participants agreed that they feel discriminated against by either mistreatment or negative judgment by healthcare providers if they disclose their sexual identity (68.8%), whereas half (50.2%) agreed that they feel embarrassed when peers (e.g., friends regardless of sexual orientation) discover that they are getting tested for HIV. Notably, there was a significantly increased likelihood of being willing to use HIVST based on their sexual orientation (*p* = 0.001), ethnicity (*p* = 0.001), engagement in anal sex with another man in the last 6 months (*p* = 0.009), prior HIV testing (*p* = 0.049), and previous use of an HIVST kit (*p* < 0.001).

**Table 1:** Participant characteristics of Malaysian MSM, stratified by willingness to use HIV self-testing kit (2019, *N* = 544)

Variables	Entire Sample *(N = 544)*		Willing to use HIVST (***n*** = 405) ^i^*n (%)*	***p***
	Frequency	%		
**Characteristics of participants**				
Age (years): Mean (±SD) ^a^	30.6 (±8.0)		30.2 (±7.6)	0.008
Sexual orientation				0.001
Homosexual/Gay/PLU^b^	432	79.4	320 (58.8)	
Bisexual	98	18.0	80 (14.7)	
Others^c^	14	2.6	5 (0.9)	
Ethnicity				0.001
Malay	272	50.0	220 (40.4)	
Chinese	180	33.1	117 (21.5)	
Other^d^	92	16.9	68 (12.5)	
Relationship status				0.425
Single^e^	409	75.2	308 (56.6)	
Married/Partnered	135	24.8	97 (17.8)	
Highest educational level				0.744
Secondary education and below	70	12.9	51 (9.4)	
Tertiary education (College/University)	474	87.1	354 (65.1)	
Employed				0.727
No	155	28.5	117 (21.5)	
Yes	389	71.5	288 (52.9)	
Monthly Income (MYR)^f^				0.158
< 3000	225	41.4	162 (29.8)	
3000 - 5999	189	34.7	150 (27.6)	
≥ 6000	130	23.9	93 (17.1)	
**Sexual Behavior Characteristics**				
Any anal intercourse with another man (last 6 months)				0.009
No	87	16.0	55 (10.1)	
Yes	457	84.0	350 (64.3)	
Condomless anal sex (last 6 months)	*n = 457*			0.190
No	93	20.4	76 (16.6)	
Yes	364	79.6	274 (60.0)	
Average number of male sexual partners (last 6 months): Mean (±SD)^a^	5.1(±12.1)		5.3(±13.6)	0.117
Ever engaged in transactional sex^g^	*n = 105*			0.420
No	483	88.8	357 (65.6)	
Yes	61	11.2	48 (8.8)	
Ever used recreational drugs before or during sex^h^				0.505
No	422	77.6	317 (58.3)	
Yes	122	22.4	88 (16.2)	
**Prior HIV testing**				
Ever been tested for HIV				0.049
No	161	29.6	129 (23.7)	
Yes	383	70.4	276 (50.7)	
Previously tested for HIV	*n = 383*			0.231
Less than 6 months ago	245	64.0	174 (45.4)	
6–12 months ago	66	17.2	53 (13.8)	
Over 12 months ago	72	18.8	49 (12.8)	
Ever used an HIV self-testing kit				< 0.001
No	407	74.8	283 (52.0)	
Yes	137	25.2	122 (22.4)	
**Stigma and Discrimination**				
I feel embarrassed when people relate HIV as “LGBT” disease	339	62.3	262 (48.2)	0.051
I feel embarrassed when peers discover that I am getting tested for HIV	273	50.2	223 (41.0)	< 0.001
I feel discriminated of being mistreated and judged by the healthcare providers if they know my sexual identity	374	68.8	292 (53.7)	0.004

Note: ^a^ SD: standard deviation; ^b^ PLU: people like us; ^c^ Queer, heterosexual, and others; ^d^ Includes Indian, Sabahan, Sarawakian, and mixed; ^e^ Includes divorced and widowed; ^f^ MYR: Malaysian Ringgit; ^g^ Received any things or opportunities (e.g., mobile phone, cash, clothes, bag, study or employment opportunity) in exchange for sex; ^h^ Includes crystal meth/"ice”, ketamine, ecstasy, poppers, GHB/GBL) before or during anal sex: ^i^ HIVST: HIV self-testing

### Conjoint analysis

Table 2 presents the acceptability scores of each HIVST service delivery scenario. The acceptability ranged from 44.9 to 77.1%, with overall mean acceptability of 56.2%. The top-rated HIVST service delivery scenario included no cost (free), anonymous (name not required to receive HIVST kit), 99–100% effective, home-delivered, finger stick (saliva-based), and the ability to receive pre-test counseling support anonymously.

**Table 2:** Acceptability (Mean) of hypothetical HIV self-testing with different attributes in order of decreasing acceptability among Malaysian MSM (2019, N = 544)

Acceptability (Mean)	HIV Self-Testing Attributes					
	Cost	Kit Collection Site	Privacy	Testing Support	Accuracy (%)	Kit Type
77.09	Free	Home delivered	Name not required	Telephone Hotline/Texting	99–100	Fingerstick
62.37	Free	Home delivered	Name required	Online videos	99–100	Oral swab
59.20	Free	At a pharmacy	Name not required	Online videos	95	Oral swab
57.15	Pay some money	At a pharmacy	Name not required	Online videos	99–100	Fingerstick
52.93	Pay some money	Home delivered	Name not required	Telephone Hotline/Texting	95	Oral swab
48.90	Free	At a pharmacy	Name required	Telephone Hotline/Texting	95	Fingerstick
47.41	Pay some money	At a pharmacy	Name required	Telephone Hotline/Texting	99–100	Oral swab
44.91	Pay some money	Home delivered	Name required	Online videos	95	Fingerstick

**Fig. 2:**
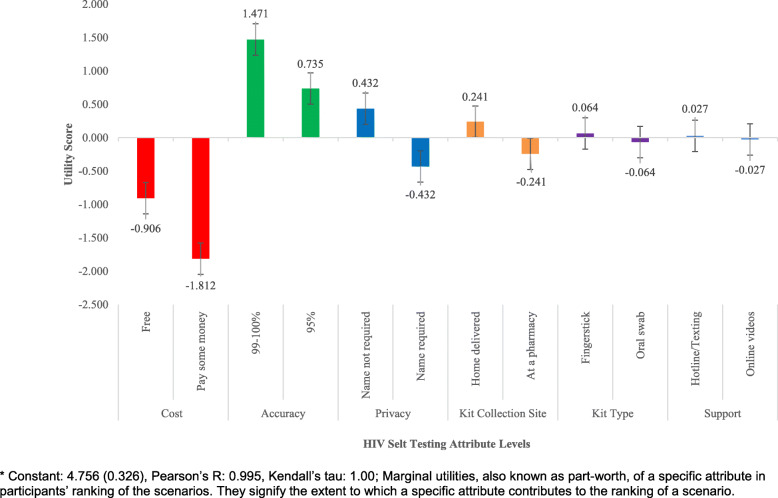
Marginal utilities of HIV self-testing attributes’ levels among Malaysian MSM (2019). * Constant: 4.756 (0.326), Pearson’s R: 0.995, Kendall’s tau: 1.00; Marginal utilities, also known as part-worth, of a specific attribute in participants’ ranking of the scenarios. They signify the extent to which a specific attribute contributes to the ranking of a scenario

Table 3 presents the marginal utility of each attribute on overall acceptability. The findings show that the cost associated with HIVST was the single most important attribute. Our sample reported higher acceptability if the HIVST kit was offered free of charge (*MUS* = -0.906), compared to paying for the kit (*MUS* = − 1.812), yielding a net RIS of 19.30. Privacy had the second-greatest impact on acceptability with an overall RIS of 18.41. Participants preferred the anonymous HIVST program (*MUS* = 0.432) to the one which requires providing a name to receive the HIVST kit (*MUS* = − 0.432). Accuracy of HIVST had the third-greatest impact on HIVST acceptability. Participants reported higher acceptability for HIVST when it was 99–100% effective (*MUS* = 1.471) compared with 95% effective (*MUS* = 0.735), yielding a RIS of 17.33. Kit collection site (*RIS* = 16.99), kit type (*RIS* = 15.86), and support (*RIS* = 12.08) had relatively low influence on HIVST acceptability. Compared to buying it over the counter (*MUS* = − 0.241)*,* participants preferred having it delivered to their home (*MUS* = 0.241). Participants preferred fingerstick HIVST kit (*MUS* = 0.064) over oral swab testing (MUS = − 0.064). Receiving testing and counseling anonymously (telephone hotline or WhatsApp texting (*MUS* = 0.027) rather than using online videos (*MUS* = − 0.027) was marginally preferred (Table 3 and Fig. 2).

**Table 3:** Relative importance and marginal utilities of HIV self-testing attribute levels among Malaysian MSM (2019)

Attributes	Attribute Levels	RIS^a^
Cost	Free	19.30
	Pay some money	
Privacy	Name not required	18.41
	Name required	
Accuracy	99–100%	17.33
	95%	
Kit Collection Site	Home delivered	16.99
	At a pharmacy	
Kit Type	Fingerstick	15.86
	Oral swab	
Testing Support	Telephone hotline / Texting	12.08
	Online videos	

Note: ^a^ Relative importance score

## Discussion

To the best of our knowledge, this is the first study to assess the acceptability of HIVST using CBA among MSM that quantifies key attributes associated with HIVST acceptability in this key population. Key findings from this study was that MSM had suboptimal rates of recent HIV testing (45.0% in the past six months), and the CBA provided useful insights as Malaysia struggles to improve HIV testing in the country. This study further offers important insights into the potential use of HIVST by MSM in Malaysia. Although the minority of our sample had previously used the HIVST, we observed a high willingness to use HIVST, a finding observed elsewhere in MSM [29–31]. Given the low current HIV testing rates in this group with a high need for testing and concomitant linkage to prevention and treatment services, innovations in HIV testing, including HIVST, may fill an important gap in HIV prevention and treatment.

Using the CBA, we ranked the weighted importance of HIVST attributes among Malaysian MSM, who remain highly stigmatized and marginalized from existing HIV prevention services. The findings revealed significant variations in participants’ attitudes and preferences of HIVST service delivery approaches that collectively or individually may strengthen efforts to scale-up HIVST in the Malaysian context. HIVST acceptability exceeded 60% for two case scenarios (6 and 4). Three key attributes were central to both scenarios – no cost, testing anonymity, and high efficacy – with other attributes (kit collection site, kit type, and counseling support) varying between the two scenarios. These attributes collectively influenced ~ 55% of all stated preferences. One discrete choice experiment (DCE) in China assessed HIV testing preferences generally (not just HIVST) among MSM and has some findings that are consistent with ours. Their study showed the two key attributes to HIV testing similar to ours and included no cost and testing anonymity [32].

As noted, the cost is the most critical attribute for MSM when considering options for HIVST service delivery. It is not surprising that access to free HIVST (fully subsidized) dominated the individual program attributes, especially given that almost half of the participants in this sample (46.8%) preferred spending less than RM20 (~USD 5) as the maximum price they would pay for one unit of HIVST kit. When cost is factored into most CBA assessments, it typically dominates since people generally appear to prefer not to pay for services that they feel should be covered by the government, health insurance, or other funders [19, 20, 32, 33]. As the market of HIVST evolves, this finding may alter as MSM gain experience with HIVST, especially as donor agencies and governments make decisions on how much to subsidize the prices for the HIVST, if at all.

Importantly, participants preferred to access HIVST kits anonymously and have it delivered to home. Findings here indicate that HIVST may have limited appeal to Malaysian MSM if anonymity is not maintained. This aligns with the results from prior studies with key populations in other geographical settings, where participants reported privacy concerns as a barrier to HIV testing in general (not necessarily HIVST) [32, 34]. Though not explored here, these findings point to the perceived high levels of stigma related to being a sexual minority, as observed in this sample. In Malaysia, where anal sex between two consenting adults is criminalized, MSM bear the dual burden of social stigma and discrimination. Consequently, MSM avoid going to hospitals/clinics for fear of disclosing their sexual identity, which entails stigma and discrimination. As a discreet and convenient approach, HIVST might be most useful in reaching MSM who are reluctant or unable to access traditional venue-based HIV testing services because of concerns about privacy, stigma, and discrimination, and, in some instances, criminalization [8, 35, 36]. Given the social costs associated with disclosing MSM identities and behaviors in Malaysia, it is important for policymakers to take into account that the appeal of HIVST will be severely diminished if MSM are required to provide their name to access HIVST.

While HIVST, when done anonymously, provides an added layer of protection and addresses stigma for MSM, it may leave potential gaps in the HIV treatment and prevention cascades, since testing provides a key step in linkage to either confirmatory testing and associated HIV care followed by ART prescription if test results are positive and to pre-exposure prophylaxis (PrEP) for high-risk MSM without HIV [12, 37–39]. Especially in settings where high levels of stigma toward MSM exist, like in Malaysia, innovative strategies are needed to complement anonymous HIVST delivery with support during testing, accurate interpretation of results, and creative solutions for expeditiously linking MSM to post-test counseling and care. Mobile technology (e.g., smartphone app: *HIVSmart!, WeTest*) is feasible and acceptable in MSM to promote HIVST and linkage to care following the self-testing, but only when testing was confidential and not anonymous [40, 41]. In our sample, anonymity was the highest preference attribute for HIVST after cost. One potential strategy to balance anonymity in HIVST with adequate linkage to care was learned in the Social Entrepreneurship Model (SET), which allowed anonymous HIV testing, but did so by asking Chinese MSM to pay money upfront (USD 23) to receive their HIVST kit, but refunded after sharing test results via a web portal) [42]. Because cost was the most important attribute in our sample, it is unsure how MSM in the Malaysian context would be willing and/or able to put money upfront for HIVST. One might consider free HIVST, but if kits were delivered to home addresses, it may not be fully anonymous. Alternatively, if they have it delivered to an alternative address, such MSM would need the motivation to provide personal information if they knew that the benefit of linkage to care through disclosure outweighed the risks. Therefore, additional research is needed to identify new strategies that balance cost and anonymity to impact HIV testing, followed by effective linkage to care.

Our data further indicated accuracy as an important attribute, with 99–100% accuracy being the preferred alternative, as expected. Previous studies have shown that concerns about the accuracy of HIVST as being one of the major barriers to HIVST uptake [8, 43], yet evidence suggests that currently approved HIVST kits have sensitivity and specificity of 99% or higher [44]. Furthermore, participants expressed a strong preference for blood-based tests using fingerstick (66.7% vs. 19.7%), although the saliva-based kit is the only HIVST kit prequalified by WHO [45]. Our study identified a preference but did not explicitly say they would not accept saliva-based testing. The preference for the blood-based tests may be due to its greater perceived accuracy and fewer false-negative test results, as compared to saliva-based tests, as suggested in prior literature [8, 46–48]. in the absence of a lived experience and personal testimonies of one method over another, it can be challenging to give credence to this preference without further exploration. Given the hypothetical nature of the scenarios presented to the participants, future studies need to verify this finding to facilitate developing the most preferred HIVST service delivery model for this population. The emergence in recent years of several new HIVST methods and technologies with increased accuracy may contribute to increasing consistent testing among this group.

Our sample generally preferred virtual interaction (telephone hotline or WhatsApp texting) for counseling support rather than in-person interactions. This preference is supported by the high levels of stigma in MSM and is supported by their preference to maintain anonymity. Moreover, this population of MSM are young and generally prefer technology-based interactions. This method also provides the users with technical support, counseling, and referrals for further HIV testing services, HIV prevention, care and treatment, and other services, including psychosocial, legal support, and violence support, as demonstrated in prior research [19, 49, 50]. This finding, along with the evidence that a large proportion of Malaysian MSM have access to smartphones [10], emphasizes the potential for a telephone hotline or smartphone app-based (e.g., WhatsApp message) platform for counseling. This finding aligns well with prior research [10], indicating the need for innovative approaches, such as mHealth, to scale-up HIVST in this highly stigmatized group.

Our data further indicated that participants were willing to make trade-offs to have the HIVST approach they prefer. For example, participants were willing to use the oral HIVST kit and use online videos for counseling in exchange for not having to disclose their name while receiving the kit. In other instances, participants were willing to pay out-of-pocket and use oral HIVST kit to avoid disclosing their identity while buying the HIVST kit. Although the traditional venue-based HIV testing models have a high impact on HIV testing, we found that low-cost with home delivery HIVST distribution model that allows for anonymous testing may be an important strategy to increase HIV self-testing in key populations that would not otherwise test, such as MSM. Much has been learned from HIVST demonstration programs targeting MSM in the other context [23, 49, 51–53], and many such lessons, as well as the user stated-preference information from the current study, might be taken into account into the decision-making process to optimize HIVST distribution program among MSM in the Malaysian setting.

Several study limitations must be acknowledged. First, this survey involved CBA but did not incorporate a segmentation analysis. Future studies should better understand that there might be subgroups of MSM who may have different patient-stated preferences. For example, in Ukrainian MSM, younger MSM and those with substance use disorders preferred PrEP delivery methods that differed from their counterparts [54]. Second, this analysis excluded TGW, and the findings should not be generalized for this key population. Further studies of patient preferences for HIVST in TGW should be conducted, even some general information is already available [55]. Third, although a brief explanation about HIVST and its attributes was provided, we do not know the extent to which participants understood every attribute (e.g., cost, privacy, accuracy, kit collection site, kit type, and testing support) while ranking the HIVST program scenarios. This is especially true since there is limited experience and no real testimonials to drive patient preferences. Third, our use of self-report measures may have resulted in participant underreporting or inconsistent reporting (e.g., HIV status) of socially undesirable behaviors. Last, and importantly, patient preferences and intentions may not fully be aligned with their practices, suggesting the need to link uptake of HIVST after stating their preferences. Further research is thus warranted to assess the impact of these issues on HIVST acceptability among our sample.

Malaysia has yet to establish guidelines for the use of HIVST. The findings from this study underscore the need to consider participant preferences and key attributes while selecting service delivery models and support tools. This could be relevant as the market for HIVST takes shape in the country, with the Malaysian Ministry of Health in the process of developing a regulatory framework for ensuring the quality of kits, as well as policies supporting safe use while broader implementation under national AIDS programs. Important.

## Conclusions

HIVST offers an alternative to traditional, venue-based testing that could significantly improve HIVST uptake and is ideally situated where stigma related to key populations, HIV, or both are prevalent. Since a number of factors are central to HIVST delivery programs, users’ preferences can play an important role when introducing such programs. In the current study, we used the stated-preference approach to explore the acceptability of HIVST distribution strategies based on a number of known attributes in Malaysian MSM. Key findings include high levels of acceptability if HIVST distribution programs are optimally organized to accommodate user preferences, notably low-cost models that ensure user anonymity. Findings are useful to guide future HIVST implementation strategies to improve the HIV treatment and prevention cascade in MSM in Malaysia.

## Data Availability

Datasets used and/or analyzed during the current study are available from the corresponding author on reasonable request.

## Abbreviations

CBA: Conjoint-based analysis
DCE: Discrete choice experiment
HIVST: HIV self-testing
LGBT: Lesbian, gay, bisexual, and transgender
MSM: Men who have sex with men
MUS: Marginal utility score
PrEP: Pre-exposure prophylaxis
RIS: Relative importance score
WHO: World Health Organization
